# Decreased expression of miR-29 family associated with autoimmune myasthenia gravis

**DOI:** 10.1186/s12974-020-01958-3

**Published:** 2020-10-08

**Authors:** Mélanie A. Cron, Cloé A. Payet, Odessa-Maud Fayet, Solène Maillard, Frédérique Truffault, Elie Fadel, Julien Guihaire, Sonia Berrih-Aknin, Adrian Liston, Rozen Le Panse

**Affiliations:** 1 Center of Research in Myology, Sorbonne University, INSERM, Association Institute of Myology, UMRS 974 Paris, France; 2 Marie Lannelongue Hospital, Paris-Sud University, Le Plessis-Robinson, France; 3 VIB Center for Brain and Disease Research, KU Leuven-University of Leuven, Leuven, Belgium; 4 Laboratory of Lymphocyte Signalling and Development, The Babraham Institute, Babraham Research Campus, Cambridge, CB22 3AT UK

**Keywords:** microRNAs, DICER, Thymus, Thymic epithelial cells, Experimental autoimmune myasthenia gravis, Interferon-β, Th17 cells, BAFF

## Abstract

**Background:**

Myasthenia gravis (MG) is a rare autoimmune disease mainly mediated by autoantibodies against the acetylcholine receptor (AChR) at the neuromuscular junction. The thymus is the effector organ, and its removal alleviates the symptoms of the disease. In the early-onset form of MG, the thymus displays functional and morphological abnormalities such as B cell infiltration leading to follicular hyperplasia, and the production of AChR antibodies. Type-I interferon (IFN-I), especially IFN-β, is the orchestrator of thymic changes observed in MG. As Dicer and miR-29 subtypes play a role in modulating the IFN-I signalization in mouse thymus, we investigated their expression in MG thymus.

**Methods:**

The expression of DICER and miR-29 subtypes were thoroughly investigated by RT-PCR in human control and MG thymuses, and in thymic epithelial cells (TECs). Using miR-29a/b-1-deficient mice, with lower miR-29a/b-1 expression, we investigated their susceptibility to experimental autoimmune MG (EAMG) as compared to wild-type mice.

**Results:**

DICER mRNA and all miR-29 subtypes were down-regulated in the thymus of MG patients and DICER expression was correlated with the lower expression of miR-29a-3p. A decreased expression of miR-29 subtypes was similarly observed in MG TECs; a decrease also induced in TECs upon IFN-β treatment. We demonstrated that miR-29a/b-1-deficient mice were more susceptible to EAMG without higher levels of anti-AChR IgG subtypes. In the thymus, if no B cell infiltration was observed, an increased expression of *Ifn-β* associated with *Baff* expression and the differentiation of Th17 cells associated with increased expression of *Il-6*, *Il-17a* and *Il-21* and decreased *Tgf-β1* mRNA were demonstrated in miR-29a/b-1-deficient EAMG mice.

**Conclusions:**

It is not clear if the decreased expression of miR-29 subtypes in human MG is a consequence or a causative factor of thymic inflammation. However, our results from the EAMG mouse model indicated that a reduction in miR-29a/b1 may contribute to the pathophysiological process involved in MG by favoring the increased expression of IFN-β and the emergence of pro-inflammatory Th17 cells.

## Introduction

MG is a neuromuscular disease characterized by invalidating muscle weaknesses. It is caused by autoantibodies targeting components of the neuromuscular junction, such as the acetylcholine receptor (AChR) [1]. In early-onset MG, the thymus is the effector organ and its removal alleviates the symptoms of the disease. The MG thymus displays functional and morphological abnormalities characterized by abnormal B cell infiltration leading to follicular hyperplasia and the production of anti-AChR antibodies [1]. These data indicate that disordered thymic processes underlie MG; however, the molecular regulators of this dysfunction remain to be elucidated. A thymic overexpression of interferon (IFN)-β and IFN-I-induced genes is observed in MG, even long after disease onset, and IFN-β seems to be the main orchestrator of thymic changes [2–4]. Besides, a causative role of IFN-I is supported by a mouse model where injections of Poly(I:C) induce thymic changes that lead to an anti-AChR response [3, 4].

Papadopoulou et al. demonstrated that miRNAs are essential to protect thymic architecture. Using conditional knock-out mice for Dicer in thymic epithelial cells (TECs, *Foxn1*^*Cre*^ *Dicer*^*fl/fl*^), they observed a premature involution and the appearance of epithelial voids with dense foci of B cells. Besides, Dicer-deficient mice are hypersensitive to Poly(I:C) in line with increased expression of Ifnar1 in TECs. This latter effect is mediated by the miR-29a/b-1 cluster, as miR-29a targets Ifnar1 and increases the sensitivity to Poly(I:C) due to an increased expression of Ifnar1 in TECs [5].

These observations suggest a link between miR-29a and the IFN-I signature observed in MG thymus. Consequently, we investigated the expression of DICER and miR-29 subtypes in the thymus of MG patients and investigated the sensitivity of miR-29a/b-1-deficient mice to experimental autoimmune MG (EAMG).

## Methods

### Human thymic samples

Thymic biopsies from early-onset MG patients (*n* = 12, 15–35 years old) were collected after thymectomy and control thymic biopsies (*n* = 6, 15–33 years old) were collected from donors undergoing cardiovascular surgery at the Marie Lannelongue Surgical Center (Le Plessis-Robinson, France). The degree of thymic follicular hyperplasia in MG patients was assessed by pathologists (high degree of hyperplasia (MH patients (*n* = 6)) with 3 or more germinal centers per section vs. low degree of hyperplasia (ML patients (*n* = 6)) with 2 or less germinal centers per section). MG patients were only treated with cholinesterase inhibitors and had no other known diseases including thymoma; all their characteristics are detailed in Table 1. All the studies on thymuses were approved by a local ethics committee (CPP, authorization number ID RCB 2010-A00250-39) and informed consent forms have been collected.

**Table 1:** Characteristics of patients whose thymus were used for RT-PCR experiments

Patient ID	Gender	Age (years)	Degree of thymic hyperplasia (1)	Interval onset - thymectomy (months)	MGFA score at thymectomy (2)	Corticoid treatment	Cholinesterase inhibitors	Anti-AChR titer (nmol/L)
MG1	F	15	Low	13	III b	No	Yes	3.45
MG2	F	23	Low	7	II a	No	Yes	2118.7
MG3	F	29	Low	7	I a	No	NS	83.7
MG4	F	35	Low	24	IV a	No	Yes	11.1
MG5	F	20	Low	6	II a	No	Yes	> 100
MG6	F	32	Low	6	II b	No	Yes	17.3
MG7	F	30	High	2	IV a	No	NS	> 100
MG8	F	30	High	14	I	No	NS	3180.2
MG9	F	25	High	3	II a	No	Yes	3.21
MG10	F	28	High	4	II a	No	Yes	60.38
MG11	F	28	High	36	III a	No	Yes	9.7
MG12	F	22	High	2	III a	No	Yes	264

(1) Degree of thymic hyperplasia: low hyperplasia (with 2 or fewer GCs per section) or high hyperplasia (with 3 or more GCs per section). (2) Clinical classification according to the Myasthenia Gravis Foundation of America (MGFA)

Primary human TECs were cultured from infant thymus collected from donors undergoing cardiovascular surgery, and correspond mainly to medullary TECs as previously described [6]. TECs were seeded (1.4 × 10^5^ cells/cm^2^) in RPMI-5% horse serum for 24 hours. Next, TECs were treated with IFN-β 1000 UI/ml (11415, R&D Systems, Lille, France) in RPMI-0.5% horse serum for 24 h.

### Animals

Thymic miR-29a/b-1-deficient mice on the B6 background were designed as previously described [5]. They were decontaminated upon arrival by embryo transfer in C57BL/6j mice purchased at Janvier Labs (Saint-Berthevin, France). Afterward, mice were bred and host in a specific pathogen-free animal care facility (Centre d’Expérimentation Fonctionelle, Sorbonne Université, Paris, France) according to European and French ethics agreements (n° 2569.01).

### RT-PCR for miRNAs on human thymic biopsies

Total RNA was extracted from human thymic biopsies using the mirVana miRNA Isolation Kit. Biopsies were first lysed in the lysis/binding buffer provided in the mirVana kit using the FastPrep FP120 instrument (Qbiogen, Illkirch, France). RNA integrity was assessed on a Bioanalyzer 2100 (Agilent Technologies, Les Ulis, France).

miRNAs were retro-transcribed from total RNA using the TaqMan MicroRNA Reverse Transcription Kit and following the Custom RT Pool protocol (ThermoFisher Scientific, Villebon-sur-Yvette, France). Briefly, RT primers were pooled with RNA samples so that the miRNAs expression could be assessed in a unique sample. qPCR reactions were carried out using the TaqMan Universal Master Mix II, no UNG (Life Technologies) on a LightCycler 480 (Roche, Meylan, France). PCR settings were as follows: 1 cycle of polymerase activation and denaturation at 95 °C for 10 min, 45 cycles of amplification at 95 °C for 15 s and 60 °C for 1 min. miRNA expression was normalized to 28S. Primers used for qPCR are listed in Table 2.

**Table 2:** List of TaqMan microRNA assays used for custom RT pool and qPCR on miRNAs

Assay ID	miRNA name	miRBase ID	Sequence
002112	hsa-miR-29a-3p	MIMAT0000086	UAGCACCAUCUGAAAUCGGUUA
002447	hsa-miR-29a-5p	MIMAT0004503	ACUGAUUUCUUUUGGUGUUCAG
000413	hsa-miR-29b-3p	MIMAT0000100	UAGCACCAUUUGAAAUCAGUGUU
002165	hsa-miR-29b-1-5p	MIMAT0004514	GCUGGUUUCAUAUGGUGGUUUAGA
002166	hsa-miR-29b-2-5p	MIMAT0004515	CUGGUUUCACAUGGUGGCUUAG
000587	hsa-miR-29c-3p	MIMAT0000681	UAGCACCAUUUGAAAUCGGUUA
001818	hsa-miR-29c-5p	MIMAT0004673	UGACCGAUUUCUCCUGGUGUUC

### RT-PCR for mRNAs

Total RNA from human thymus was obtained as described above. Total RNA from mouse thymus or from TEC cultures were extracted in TRIzol (ThermoFisher Scientific) using the FastPrep FP120 instrument (Qbiogen, Illkirch, France) for thymic biopsies. RNA (1 μg) was reverse-transcribed for 1 h at 42 °C using AMV (Ref 10109118001, Roche Life Science, Meylan, France) with oligo-dT (ThermoFisher Scientific). PCR reactions were carried out with the LightCycler 480 SYBR Green Master Mix on the LightCycler® 480 System (Roche). All samples were normalized to 28S or GAPDH. The primer sequences (Eurogentec, Angers, France) are listed in Table 3.

**Table 3:** List of primers used for RT-PCR on mRNAs

	Gene name	Sens primer	Antisens primer
**Human primers**	*28S*	GGTAGGGACAGTGGGAATCT	CGGGTAAACGGCGGGAGTAA
	*CD19*	TACTATGGCACTGGCTGCTG	CACGTTCCCGTACTGGTTCT
	*DICER*	TGCTGAAACTGCAACTGACC	CAGGGTCCCAGAACTACCAA
	*GAPDH*	CGACCACTTTGTCAAGCTCA	AGGGGTCTACATGGCAACTG
	*IFNAR1*	CCTCCTGTGAGCCTAAGTGC	AAGGGCCTACCCTCAGTGTT
**Mouse primers**	*Baff*	TTCCATGGCTTCTCAGCTTT	GGAATTGTTGGGCAGTGTTT
	*Ccl21*	CCCTGGACCCAAGGCAGT	AGGCTTAGAGTGCTTCCGGG
	*Cd19*	CCCTCACCTTCGAGTTTCTG	TAGGTTCACAGGTCCCAAGG
	*Cxcl13*	TGAGGCTCAGCACAGCAA	ATGGGCTTCCAGAATACCG
	*Cxcr5*	ATGGCCTTAATGTGCCTGTC	CTTCTGGAACTTGCCCTCAG
	*Gapdh*	AACTTTGGCATTGTGGAAGG	ACACATTGGGGGTAGGAACA
	*Ifn-β*	CCCTATGGAGATGACGGAGA	CTGTCTGCTGGTGGAGTTCA
	*Ifn-α2*	TCTGTGCTTTCCTCGTGATG	TTGAGCCTTCTGGATCTGCT
	*Ifnar1*	CAAGTGTGCCTGGCTTGTTC	AGAGAAGTCCGAGGCCATCT
	*Ifnar2*	ACATGGGTCCTGGCTCAAAG	GGCAGAGAAAGGGTTGCTCT
	*Ifn-γ*	CAGCAACAGCAAGGCGAAA	GCTGGATTCCGGCAACAG
	*Il-4*	GGTCTCAACCCCCAGCTAGT	GCCGATGATCTCTCTCAAGTGAT
	*lL-6*	AGTTGCCTTCTTGGGACTGA	TCCACGATTTCCCAGAGAAC
	*Il-10*	GCTCTTACTGACTGGCATGAG	CGCAGCTCTAGGAGCATGTG
	*Il-17a*	TTTAACTCCCTTGGCGCAAAA	CTTTCCCTCCGCATTGACAC
	*Il-21*	GGACCCTTGTCTGTCTGGTAG	TGTGGAGCTGATAGAAGTTCAGG
	*Il-23*	CCGTTCCAAGATCCTTCGAA	GACCCGGGCTGCTATGG
	*Tgf-β1*	CAAGGGCTACCATGCCAACT	CCGGGTTGTGTTGGTTGTAGA

### Experimental autoimmune myasthenia gravis (EAMG)

Six to 8-week-old C57BL/6 male and female mice were used as inbred miR-29 a/b-1 heterozygous (HET), WT siblings on the C57BL/6 background, and additional C57BL/6j WT mice from Janvier Labs. Male and female mice were combined in the analyses, with no sex-based differences observable in the EAMG model [7]. The extraction of Torpedo Californica AChR (T-AChR) was led as previously described [8]. T-AChR was emulsified with an equal volume of Complete Freund’s adjuvant (CFA; F5881, ThermoFisher Scientific) supplemented with heat-inactivated *Mycobacterium tuberculosis* (10 mg/ml, H37RA, BD Difco, Villepinte, France). Mice were subcutaneously injected (200 μl/mouse, 30 μg AChR) at several sites (hind foot-pads, tail base and in the back). Control mice were injected similarly with CFA emulsion devoid of T-AChR. After 4 weeks, mice were immunized a second time with T-AChR emulsified in CFA only at the tail base and in the back.

The global clinical score was graded from 0 to 9 by taking into account the weight evolution, muscle strength, and the inverted grid test. Each of these measures was graded on 3, as described by Weiss et al. with minor modifications as follows [8]. The grip test measurements were done after a 5-min run in a treadmill. Grip test values were normalized on the weight of the animals since experiments were performed on female and male mice. For each mouse, the weight and grip test measures were compared to those obtained before the immunization, and a percentage representing the loss/gain of weight or of muscle strength along the experiment was determined. The grading on 3 was then made as detailed by Weiss et al. [8]. For the inverted grid test, mice were first tired by gently dragging them across a grid 20 times. Immediately after, the grid was inverted and held steadily for 1′30 ′′. During this time-lapse, mice were carefully observed to detect any signs of abnormal behavior. Mice were considered sick when they reached a global clinical score of 2. Mice reaching a global clinical score of 9 were euthanized.

### ELISA for anti-AChR antibodies

ELISA experiments were carried out on serum samples collected at sacrifice (day 43). 96-well plates were coated with 0.5 μg/mL of T-AChR diluted in 10 mM NaHCO3 buffer, pH 9.6, overnight at 4 °C. Wells were blocked with PBS 10% fetal calf serum for 2h30 at 37°C. Samples were diluted in PBS 0.2% BSA (1:100 000) and incubated at 37 °C for 1 h and 30 min. One hundred microliters of biotinylated rabbit anti-mouse IgG (1/10000, E0413, Dako, Courtaboeuf, France) or biotinylated anti-mouse IgG subtypes were added for 1 h and 30 min at 37 °C (anti-IgG2b 1/250 (553393, BD Biosciences, Le Pont de Claix, France); anti-IgG2c (1/5000, SA5-10235, ThermoFisher Scientific) or anti IgG1 (1/100; 553441, BD Biosciences). Samples were incubated 30 min with 100 μL of streptavidin–horseradish peroxidase (diluted at 1:10 000) (S911, ThermoFisher Scientific). Tetramethylbenzidine was used for color development, and the optical density at 450 nm was measured with the SPARK 10 M microplate reader (TECAN Life Sciences, Grödig, Austria). Between each step, wells were washed 4 times with 200 μl of PBS 0.05% Tween 20.

### Detection of relative affinity of anti-AChR IgG antibodies

The relative affinity of anti-AChR IgG antibodies was determined based on the above ELISA method using a potassium thiocyanate (KSCN) elution step [9]. Increasing concentrations of KSCN in 0.1 M PBS (pH 6.0) for 15 min at room temperature before the biotinylated rabbit anti-mouse IgG. KSCN concentrations ranging from 1 to 8 M were tested. As anti-AChR antibodies were totally removed at higher KSCN concentrations than 4 M of KSCN. Concentrations below 4 M of KSCN were used for linear regression in GraphPad and the relative affinity corresponds to the molarity of KSCN resulting in 50% of the absorbance obtained in the absence of KSCN.

### Immunohistochemistry

Cryosections of thymic samples (7 μm) were fixed in ice-cold acetone for 20 min and unspecific binding sites blocked with 2% BSA. Sections were stained for medullary thymic epithelial cells with a Keratin 5 polyclonal antibody (Biolegend, Ozyme, Saint-Cyr-L’école, France) and a donkey anti-rabbit IgG Alexa-Fluor-488 (ThermoFisher Scientific), while B cells were detected with a B220 biotinylated antibody (clone RA3-6B2, BD Biosciences) and streptavidin Alexa-Fluor-594 (S11227, ThermoFisher Scientific). Images were acquired with a ZeissAxio Observer Z1 Inverted Microscope. The number of B cells was counted in 6–8 fields representative of each thymic section.

### Statistical analysis

For 2-by-2 comparisons, the non-parametric Mann-Whitney test was applied as specified in figure legends. To analyze the mouse susceptibility to EAMG, the comparisons among the different mouse groups in kinetic were performed using a two-way ANOVA with Bonferroni adjustment for multiple comparisons. Correlation analyses were performed using Spearman’s correlation coefficient for non-Gaussian distributed variables, with a *p* < 0.05 considered significant.

## Results

### Decreased expression of DICER correlates with reduced miR-29a-3p expression in MG patients

**Fig. 1:**
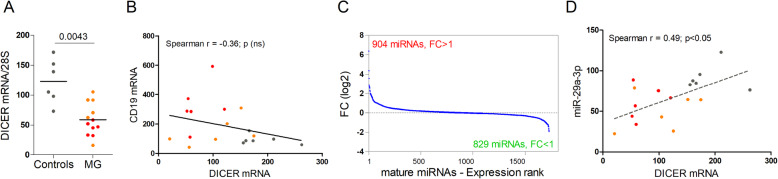
Altered DICER expression in human MG thymuses. **a** RT-PCR analysis for DICER mRNA in the thymus of non-MG adults (*n* = 6) and MG patients (*n* = 12) with a low (orange dots) and high (red dots) degree of thymic hyperplasia. Data were normalized to the 28S. *P* values were assessed by the Mann-Whitney test. **b** Correlation between DICER and CD19 mRNA expression in control (grey dots) and MG thymuses (orange and red dots) was performed using Spearman’s correlation test, with a *p* < 0.05 considered significant. **c** Using data from a thymic miRnome study [10], the distribution of FCs (fold changes) for 1733 mature miRNAs. Each FC was calculated by comparing the mean of raw intensity values for MG thymuses (*n* = 12) over the one of non-MG adult control thymuses (*n* = 6). **d** Correlation between DICER mRNA and miR-29a-3p expression in control (grey dots) and MG thymuses (orange and red dots) was performed using Spearman's correlation test, with a *p* < 0.05 considered significant

To investigate the putative role of the DICER/miR-29/IFN-I axis in MG, we analyzed the expression of DICER mRNA, a key enzyme in miRNA biogenesis. We observed a 4.6-fold decrease of expression in the thymus of MG patients, compared to healthy control thymus (Fig. 1a). Even if the DICER mRNA expression seemed lower in patients with a high degree of follicular hyperplasia (Fig. 1a), this was independent of the abnormal presence of B cells in the thymus. Indeed, no correlation between CD19 and DICER mRNA expression was observed (Fig. 1b). To assess the impact of reduced DICER expression on the global miRNA profile, we analyzed published miRnome data obtained from the same thymic biopsies [10]. Analyzing the expression of 1733 mature thymic miRNAs in MG patients as compared to controls, we observed almost an equal repartition of up- and down-miRNAs, with 904 and 829 miRNAs with a fold change (FC) higher and lower than 1, respectively (Fig. 1c). Despite no systematic impact of reduced DICER expression on the global expression of thymic miRNAs, we observed a significant correlation between DICER mRNA and miR-29a-3p expression (Fig. 1d). As Papdopoulou et al. demonstrated that DICER deletion in TECs increased sensitivity to IFN-I signalization and that miR-29a was the best candidate to mediate this effect [5, 1]. This association of reduced DICER and miR-29a expression in the MG thymus warranted further investigation as a potential causative link.

### The miR-29 family is downregulated in the thymus of MG patients

**Fig. 2:**
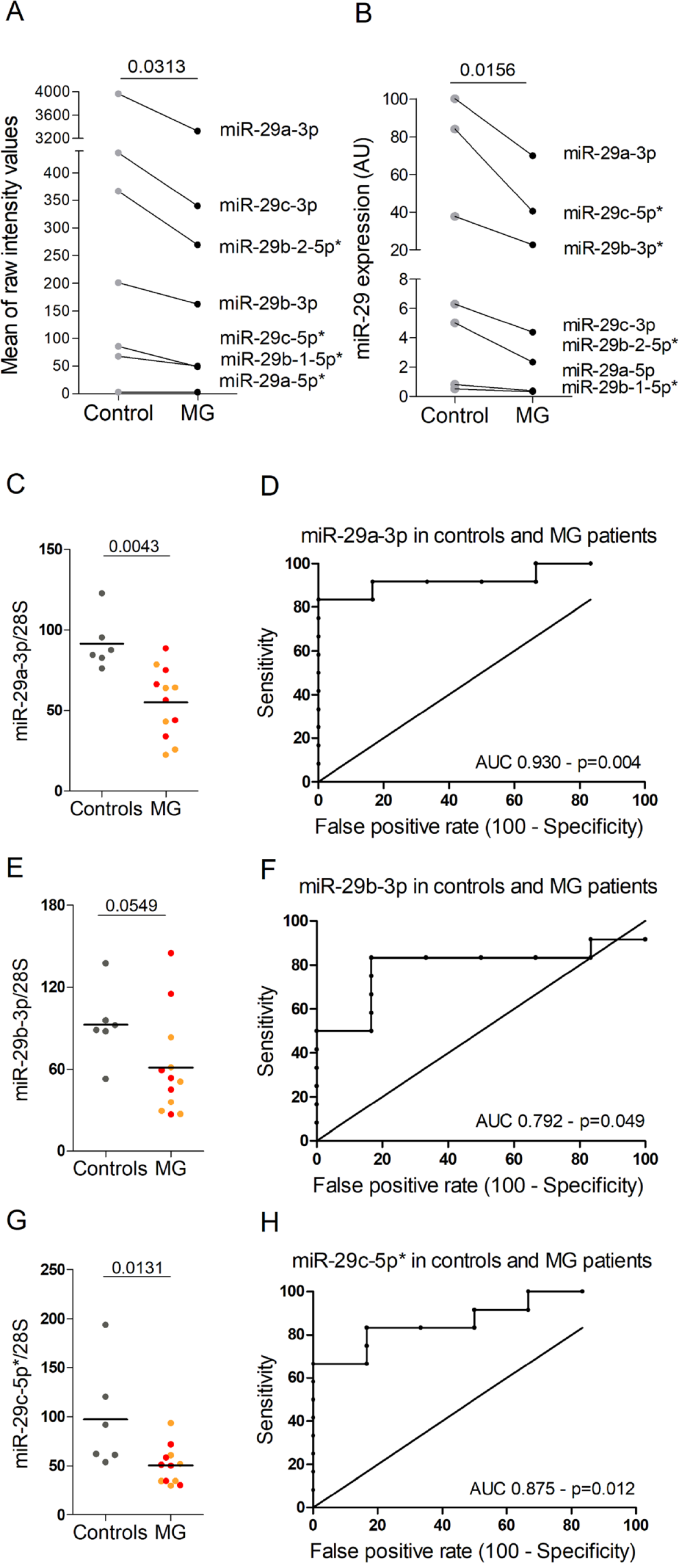
Decreased expression of miR-29 subtypes in human MG thymuses. **a** miR-29 subtypes expression was assessed using data from a thymic miRnome study [10]. Each point corresponds to the mean of raw intensity values for 6 non-MG adult control thymuses and 12 MG thymuses. *p* value was assessed by the Wilcoxon test. **b**–**h** miR-29 subtypes expression was assessed by RT-PCR in 6 non-MG adult control thymuses and 12 MG thymuses. **b** Each point corresponds to the mean values for the controls and MG thymuses. *p* values were assessed by the Wilcoxon test. **c**, **e**, **g** Detailed expression of miR-29a-3p, miR-29b-3p, and miR-29c-5p in control and MG thymuses with a low (orange dots) and high (red dots) degree of thymic hyperplasia. Data were normalized to the 28S. *p* values were assessed by the Mann-Whitney test. **d** ,**f** ,**h** Analyses of the sensitivity and specificity with ROC curves for miR-29a-3p, miR-29b-3p, and miR-29c-5p* in control and MG thymuses

The miR-29 subtypes are expressed by two genomic clusters: the miR-29a cluster (miR-29a/b-1 on chromosome 1 in human and mice) and the miR-29c cluster (miR-29b-2/c on chromosome 7 in human and chromosome 1 in mice) [11]. Using miRnome data previously published [10], we observed a significant decrease expression of all the members of the miR-29 family (miR-29a-3p, miR-29c-3p, miR-29b-2-5p*, miR-29b-3p, miR-29c-5p*, miR-29b-1-5p* and miR-29a-5p*) in MG thymuses compared to control thymuses (Fig. 2a). To validate these observations, we analyzed by RT-PCR the expression of all miR-29 subtypes. We observed that miR-29a-3p was the most strongly expressed miR-29 thymic subtype (Fig. 2a, b), as consistent with the data from the mouse thymus [5]. We confirmed that all miR-29 miRNAs were significantly downregulated in MG thymuses except the very low expressed miR-29b-1-5p* (Fig. 2b). Detailed results are showed for the three most highly expressed miRNAs, miR-29a-3p, miR-29b-3p, and miR-29c-5p* (Figs. 2c, e, g). The decreases were observed in all MG thymuses independent of the degree of thymic hyperplasia. In addition, ROC curve analyses demonstrated the high sensitivity and specificity for these three miR-29 subtypes for discriminating MG patients from controls (Fig. 2d, f, h).

These data demonstrated that miR-29 subtypes were all downregulated in the thymus of MG patients, potentially leading to a higher expression of genes targeted by miR-29 miRNAs.

### miR-29 subtypes are downregulated in TECs from MG patients

**Fig. 3:**
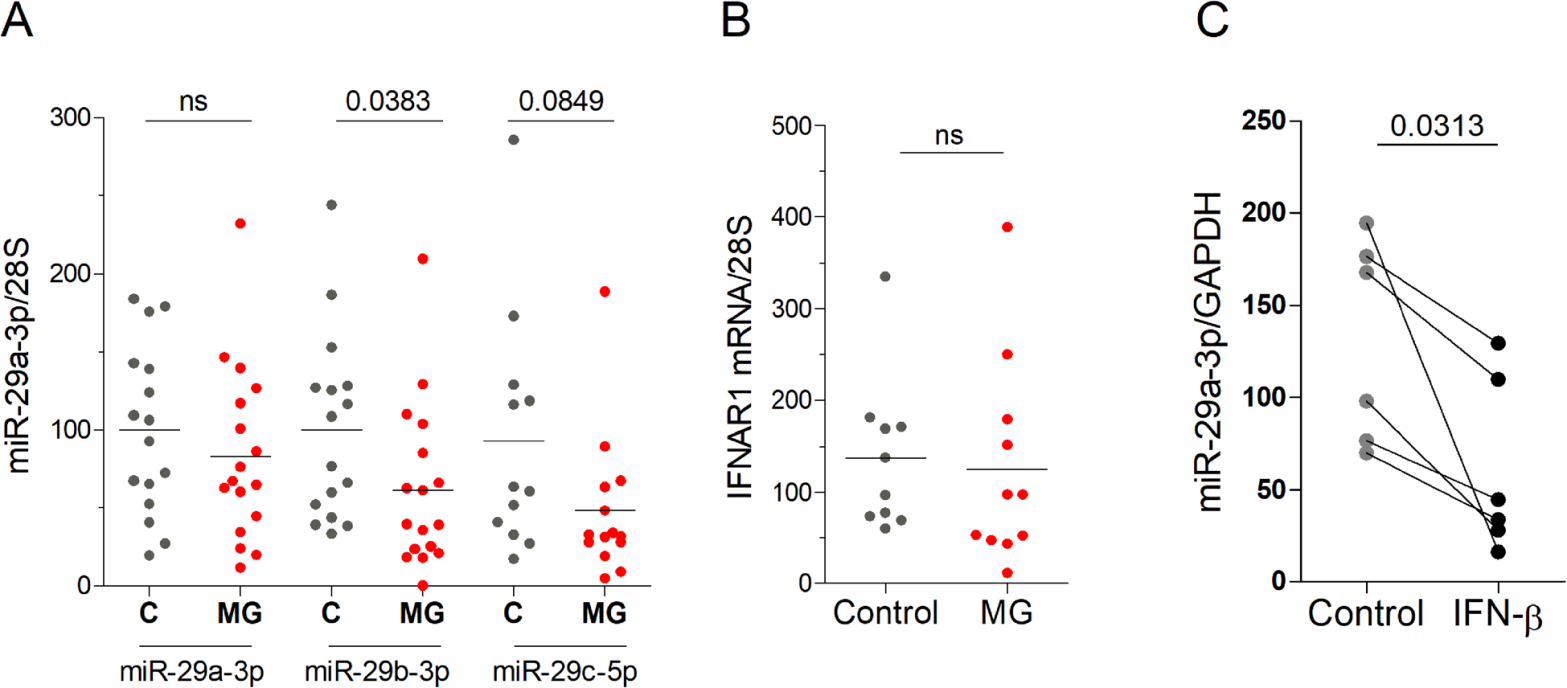
Altered expression of miR-29 subtypes in human TECs. **a** RT-PCR analyses of miR-29a-3p, miR-29b-3p, and miR-29c-5p* expressions in TECs from non-MG thymuses (*n* = 12–16) compared to MG thymuses (*n* = 14–17). **b** RT-PCR analysis of IFNAR1 in TECs from non-MG adult thymuses (*n* = 10) compared to MG thymuses (*n* = 11). Data were normalized to the 28S (**c**). TEC cultures from 6 different donors were stimulated for 24 h with IFN-β and miR-29a-3p analyzed by RT-PCR. Data were normalized to the GAPDH. *p* values were assessed by the Mann-Whitney test for **a** and **b** and by the Wilcoxon test for **c**

To determine the cellular source of miR-29 dysregulation in the MG thymus, we analyzed the level of expression of the three main thymic miR-29 subtypes in TECs derived from thymic explant cultures. We observed decreased expression or downwards trends for each of the three miR-29 subtypes tested (Fig. 3a). We also analyzed miR-29a-3p, miR-29b-3p, and miR-29c-5p* expression in thymocytes/lymphocytes freshly extracted from 5 MG and 5 control thymuses. miR-29a-3p and miR-29b-3p were expressed at 4–7-fold lower levels in thymocytes versus TECs from control donors, and miR-29c-5p* was borderline undetectable in thymocytes. No decrease expression of miR-29 subtypes was observed in thymocytes from MG patients compared to non-MG controls (data not shown), further supporting that the decreased expression in TECs could reflect what was observed at the whole thymus level. Nevertheless, the decrease in TECs was less significant than in the whole MG thymus probably as TECs were analyzed after 7 days in culture conditions that are different than the inflammatory thymic MG context.

Despite the decrease expression of the three miR-29 subtypes, no increased expression for IFNAR1 was observed (Fig. 3b). This suggests that there might be a threshold level for miR-29 to decrease IFNAR1 mRNA expression in TECs. In addition, human IFNAR1 is not listed as targeted by miR-29a according to the usual sequence matching databases (miRanda, TargetScan, and DIANA microT), and thus species differences clearly exist. A link between miR-29 and the IFN-I pathway was assessed through the exposure of TECs from healthy donors to IFN-β, as IFN-β is known to be overexpressed in the thymus of MG patients [3]. Through analyzing the effect of IFN-β on miR-29a-3p, the most highly expressed miR-29 subtype, we observed that IFN-β induced a decreased expression of miR-29a-3p (Fig. 3c). The effect of IFN-β on miR-29a-3p expression is independent of DICER as IFN-β did not affect DICER mRNA expression in TECs (data not shown). Together, these data indicate that the down-regulation of miR-29 subtypes in the thymus of MG patients could be due to a lower expression of these miRNAs in TECs, potentially mediated in response to high levels of IFN-β exposure in the MG thymus.

### miR-29a/b-1 heterozygous mice display a higher sensitivity to MG

The observation of reduced DICER and miR-29a expression in MG patient thymuses suggests a potential causative pathway for disease. Based on the known biology of miR-29a, the reduced expression could elevate response to IFN-I, escalating the disease process. Conversely, the identified expression changes in patients could be correlative, rather than causative. In order to test a potential causative link, we turned to the mouse model. Mice heterozygous for the deletion in the miR-29a/b-1 cluster (HET mice) mimic the 2-fold expression decrease observed in MG patients [5]. We, therefore, sought to compare the response of HET and wild-type (WT) control mice to the induction of MG, using the EAMG model.

**Fig. 4:**
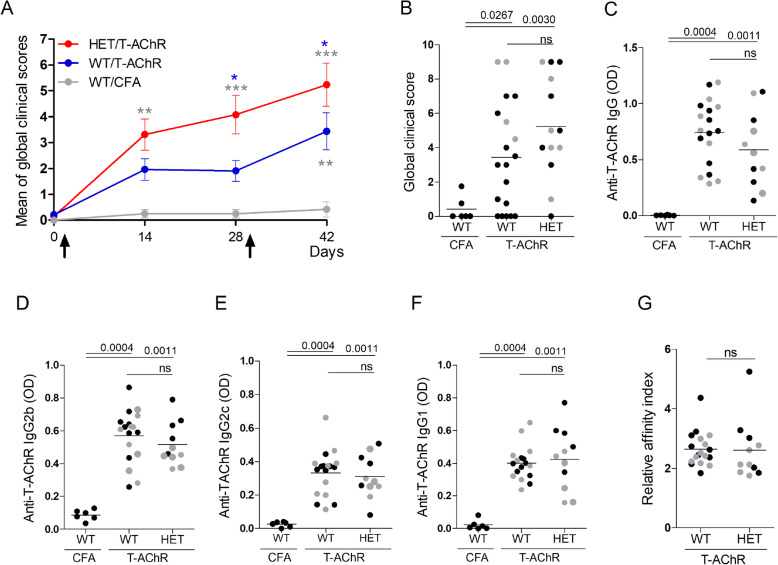
Higher susceptibility to EAMG of miR-29a/b-1 heterozygous mice. C57BL/6 WT and miR-29a/b-1 HET mice were immunized with CFA/T-AChR or just CFA (for a group of WT mice) at day 0 and boosted at day 30 (as indicated with arrows in **a**). Clinical evaluations were done at days 0, 14, 28, and 42. A global clinical score was calculated for each mouse taking into account the weight loss, the grip test, and the inverted grid test, as detailed in the method section. **a** Mean of global clinical scores was calculated for each mouse group (±SEM) and showed in kinetic. Data were analyzed by the two-way ANOVA test and *p* values determined with Bonferroni posttests. *p* values are indicated if significant with grey asterisks for WT/T-AChR and HET/T-AChR as compared to WT/CFA, and with bleu asterisks for HET/T-AChR as compared to WT/T-AChR. **b** Global clinical scores were given for each mice at the end of the experiment (day 42). **c**, **d** Anti-T-AChR antibodies were measured by ELISA and detected with anti-mouse IgG (**c**), IgG2b (**d**), IgG2c (**e**), or IgG1 (**f**) antibodies. Relative affinity index of anti-AChR IgG antibodies was determined using KSCN thiocyanate as detailed in the methods (**g**). **b**–**g** *p* values were assessed by the Mann-Whitney test. Male mice are represented with grey dots

Using a global clinical score, we observed that miR-29a/b-1 HET mice were more susceptible to EAMG induction compared to WT mice. HET mice developed symptoms more rapidly than WT mice even before the boost immunization (Fig. 4a). At the end of the experiment, 92% of HET mice were sick (with a global clinical score over 2) as compared to only 63% for the WT mice (Fig. 4b). We measured the serum levels of anti-T-AChR IgG and specifically anti-AChR IgG1, IgG2b, and IgG2c (IgG2 isotypes acting via the complement in C57BL/6 mice). All mice immunized with T-AChR developed anti-AChR antibodies that were not detected in CFA-injected control mice. However, we did not observe any differences between miR-29a/b-1 HET mice compared to WT mice (Figs. 4c–f). We also measured the levels of anti-mouse AChR IgG, IgG2b, and IgG2c by coating the ELISA plates with a mouse AChR peptide but did not detect any increased level of anti-mouse AChR antibodies in miR-29a/b-1 HET mice (data not shown). Consequently, the levels of anti-AChR antibodies did not explain the higher clinical score observed in miR-29a/b-1 HET mice. To characterize the properties of anti-AChR antibodies from WT as compared to miR-29a/b-1 HET mice, we measured the antibody affinity but did not detect any difference between the two mouse strains (Fig. 4g).

**Fig. 5:**
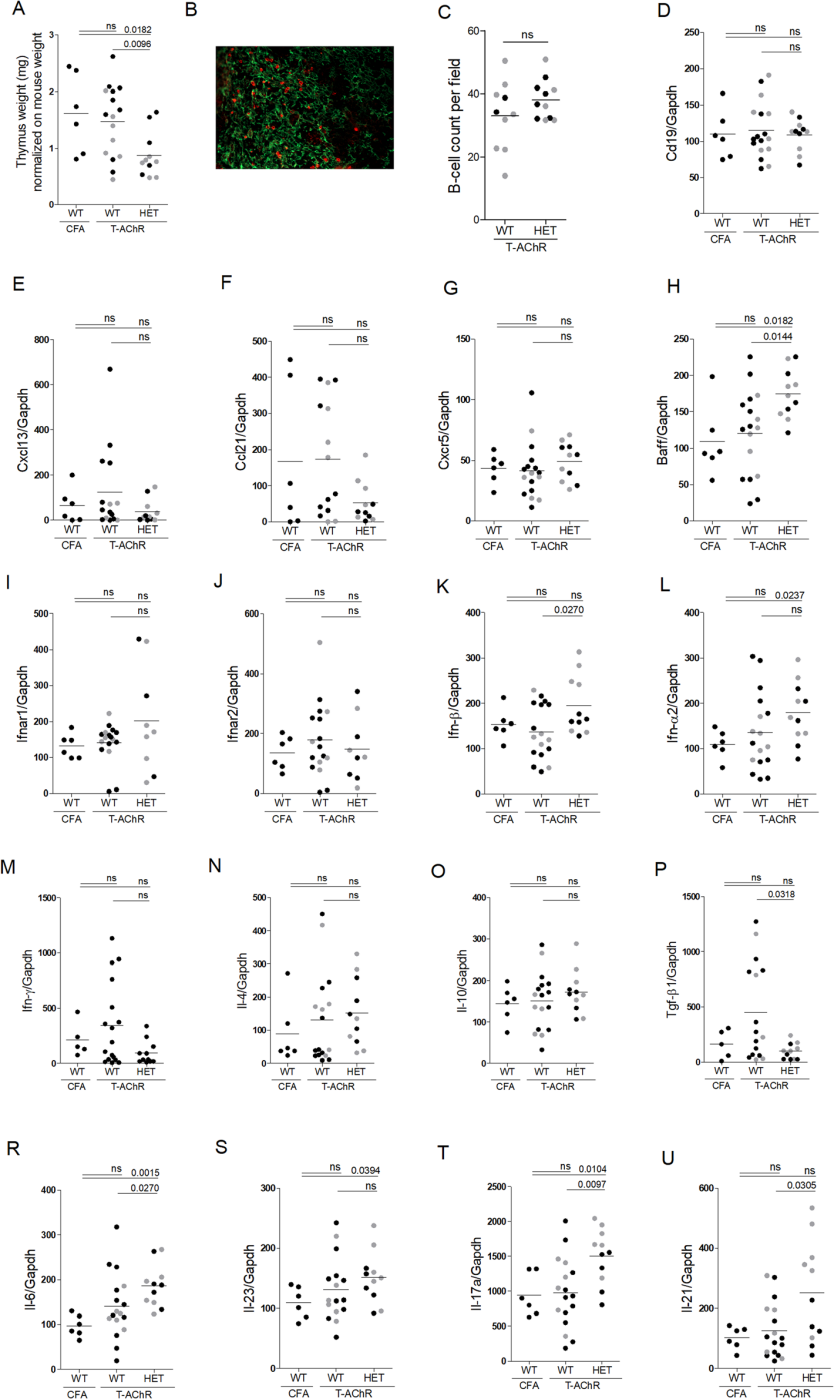
Inflammatory thymic signature in miR-29a/b-1 heterozygous mice in the EAMG model C57BL/6 WT and miR-29a/b-1 HET mice were immunized with CFA/T-AChR or just CFA (for a group of WT mice) at day 0 and boosted at day 30. Mice were sacrificed at day 43. Male mice are represented with grey dots. **a** The weight of thymuses was normalized to mouse weight. **b** Representative picture of thymus labeling for cell counting. Thymic sections were stained for TECs with a Keratin 5 antibody (green) and for B cells with a B220 antibody (red). **c** The number of B cells was counted in 6–8 fields representative of thymic sections and each point corresponds to the mean for each mice. **d**–**u** RT-PCR analyses for *Cd19*, *Cxcl13*, *Ccl21*, *Cxcr5*, *Ifnar1*, *Ifnar2*, *Ifn-β*, *Ifn-α2*, *Il-6*, *Il-23*, *Il-17a*, *Il-21*, *Ifn-γ*, *Il-4*, *Il-10*, and *Tgf-β1* mRNA expression in mouse thymuses. Data were normalized on GAPDH. *p* values were assessed by the Mann-Whitney test

Upon induction of EAMG, HET mice exhibited strong thymic involution (Fig. 5a) consistent with published results. A decrease in cellularity is observed in the thymus of miR-29 KO mice from 9 weeks old but the proportion of double-positive, double-negative, CD4, or CD8 thymocytes are not affected [5]. Unlike the published work, however, we did not detect an increase in CD19^+^ B cells in the thymus of HET mice, by either immunohistochemistry (Figs. 5b, c) or by RT-PCR (Fig. 5d). In addition, no increases were detected for *Cxcl13* or *Ccl21* mRNAs (Figs. 5e, f), two chemokines involved in B cell recruitment in MG thymus, or for *Cxcr5* mRNA (Fig. 5g), Cxcl13 receptor essential for B recruitment [12, 13]. *Baff* mRNA expression was significantly upregulated in the thymus of miR-29a/b-1 HET mice compared to WT and CFA mice (Fig. 5h).

Regarding the IFN-I signature, no increased expression in *Ifnar1* or *Ifnar2* mRNAs was observed in miR-29a/b-1 HET mice (Figs. 5i–j). However, in the thymus of miR-29a/b-1 HET mice, *Ifn-β* mRNA was significantly increased compared to WT mice (Fig. 5k) and *Ifn-α2* mRNA was also slightly increased but only compared to CFA mice (Fig. 5l). Cytokines defining T cell subsets were also analyzed by RT-PCR: *Ifn-γ* (expressed by Th1 cells), *Il-4* (expressed by Th2 cells), *Il-10* and *Tgf-β1* (expressed by Treg cell), *Il-17a* and *Il-21* (expressed by Th17 cells), *Il-6*, *Il-23* (inducing Th17 differentiation). No changes were observed for *Ifn-γ, Il-4, Il-10* (Fig. 5m, o) and a decrease was observed for *Tgf-β1* (Fig. 5p) in miR-29a/b-1 HET compared to WT mice. However, we observed a clear increased expression of *Il-17a* and *Il-21* in miR-29a/b-1 HET mice as compared to WT mice (Fig. 5r, t, u) suggesting an increase in Th17 cells. Accordingly, the expression of *Il-6* known to favor Th17 differentiation was also increased in the thymus of miR-29a/b-1 HET mice (Fig. 5r) but not that of IL-23 (Fig. 5s).

Altogether, these results demonstrate that reduced miR-29 can have a direct pathological impact on the disease MG susceptibility. This is independent of thymic B cell infiltration but could be associated with a stronger Th17 signature.

## Discussion

miRNAs are potent modulators of protein expression and are therefore involved in many physiological and pathophysiological processes. Specific miRNAs are already known to be involved in thymic pathogenesis associated with MG [10, 14–16]. Here, we investigated the implication of DICER and the miR-29 family in thymic changes in early-onset MG.

We observed a significant decreased expression of DICER in MG thymuses. Papadopoulou et al. showed the importance of Dicer in mouse thymic architecture by deleting Dicer specifically in TECs [5]. They observed a premature thymic involution, the formation of epithelial voids, and the presence of dense B cell foci. In early-onset MG, the thymus is characterized by increased presence of B cells and germinal center development [1]. However, here, we did not observe a correlation between DICER expression and the degree of follicular thymic hyperplasia or the expression of CD19 mRNA. The decreased expression of DICER in the thymus of MG patients did not seem as critical as the total deletion of Dicer in TECs in mice, as it did not lead to a global decreased expression of thymic miRNAs. However, DICER also possesses non-canonical, miRNA-independent roles [17]. DICER protects cells from cytotoxic accumulation of endogenous dsRNAs, such as Alu RNAs that can lead to abnormal activation of damage-associated molecular patterns (DAMPs) and subsequently sterile inflammation associated with IFN-I signalization [18]. Cufi et al. demonstrated that the injection of dsRNA to mice leads to thymic changes in link with IFN-I signalization and favors the development of a specific autoimmune reaction against AChR [3].

We observed a significant correlation between DICER expression and miR-29a in human MG thymuses. In light of the pathological nature of IFN-I in MG [3, 4], and the role of miR-29a in desensitizing the thymus to IFN-I [5], this suggests a putative pathological pathway. The proposed pathway between Dicer and IFN-I signaling in the mouse is via miR-29a/b-1 regulation of Ifnar1 expression in TECs. Elevated Ifnar1 in TECs would explain the increased sensitivity of miR-29a/b-1-deficient mice to pathogen-related signals, as demonstrated with the injection of Poly(I:C) [5]. Here, we observed that all miR-29 subtypes were downregulated in the thymus of MG patients. This decreased expression was also observed in TEC cultures deriving from MG thymuses and could be in link with the inflammatory status of the MG thymus, as we observed a decreased expression of miR-29a in TECs upon IFN-β treatment. In multiple sclerosis patients, a downregulation of miR-29 family is also observed in peripheral blood mononuclear cells upon IFN-β treatment [19]. Besides, a decreased expression of miR-29a has also been observed in other inflammatory conditions, such as in fibroblasts in systemic sclerosis [20], in lung and plasma in chronic obstructive pulmonary disease [21], in renal tissues in IgA nephropathy [22], in peripheral blood T cells in Hashimoto’s thyroiditis patients [23], and upon bacterial infection in IFN-γ producing cells [24].

As miR-29 subtypes were downregulated by about 50% in MG thymuses, we wondered if miR-29 HET mice were more susceptible to EAMG and displayed thymic changes as in the human disease. We demonstrated that miR-29 HET mice were more susceptible to EAMG according to the global clinical score. A decrease of thymic miR-29 miRNAs could favor the emergence of autoreactive T cells against α-AChR, as the absence of miR-29a/b-1 cluster selectively affects the Aire-dependent tissues-specific antigen expression in TECs [25]. Nevertheless, this enhanced susceptibility to EAMG does not seem due to a genetic increase in autoimmunity, as miR-29a/b-1 KO are protected in the collagen-induced arthritis (CIA) mouse model [26].

In our EAMG experiment, miR-29a/b-1 HET mice did not display higher anti-AChR antibody titers and the affinity of their anti-AChR antibodies was not altered either. Similarly, in MG patients, the severity of the disease is not correlated with the antibody titer [1], and it is correlated with the degree of thymic follicular hyperplasia [27]. However, we did not observe increased B cell infiltration and follicular hyperplasia in the thymus nor did we observe increased expression of chemokines susceptible to induce B cell recruitment. It should be noted that while the EAMG model with WT C57BL/6 mice leads to the production of anti-AChR antibodies and muscular symptoms as for MG patients, this model does not show any of the thymic abnormalities observed in the human disease [28]. In mice, a strong combination of inflammation and CXCL13 overexpression seems mandatory to initiate thymic B cell recruitment [29, 8].

It was previously published that the regulation of ifnar1 expression by miR-29a was a likely molecular mediator [5]. We did not observe an increased thymic expression of Ifnar1 either because the decrease in miR-29a/b1 in heterozygous mice was not sufficient or because mRNA analyses were done in whole thymus and not in purified TECs. Ifnar2 mRNA expression, that could also be targeted by miR-29a (www.targetscan.org), was not altered either. However, in our EAMG experiment, we showed the overexpression of thymic IFN-β mRNA in miR-29a/b1 HET as compared to WT mice. In WT C57BL/6 mice, a thymic increase in Ifn-β promotes the overexpression of chemokines such as CXCL13 and CCL21 that favor B cell recruitment [4]. It is possible that in heterozygous miR-29 mice the level of Ifn-β was not sufficient to trigger the expression of these chemokines and a subsequent B cell recruitment. It was nevertheless strongly associated with the overexpression of Baff, and the differentiation of Th17 associated with increased expression of Il-6, Il-17a, and Il-21 mRNA. BAFF expression in increased in MG thymus [4]. BAFF is known to induce the expansion of activated B cells and their survival but no increased number of B cells was detected in EAMG WT and miR-29 HET mice. Recent research works have also demonstrated that BAFF can promote T cell activation, proliferation, and differentiation, in particular for Th17 cells [30]. Zhou et al. demonstrated that constitutive overexpression of Baff in transgenic mice promotes the generation of Th17 cells and aggravates experimental autoimmune encephalomyelitis (EAE) [31]. A decrease in *Tgf-β1* was also detected in the thymus of miR-29-deficient mice in the EAMG model. Tgf-β1 plays an important role in controlling autoimmunity and abnormal inflammation by providing signals limiting immune activation and in particular suppressing Th17 cells expansion [32]. At low concentrations, Tgf-β1 synergizes with IL-6 and IL-21 favoring Th17 cell differentiation [33]. In the human MG disease, a Th17 signature is also observed in the thymus [34]. This Th17 signature could explain that miR-29a/b1 HET were more severely affected in the EAMG model.

## Conclusion

So far, most of thymic miRNAs described as dysregulated in early-onset are associated with GC development, such as miR-7, miR-24, miR-139, miR-143, miR-145, miR-146, miR-150, miR-452, miR-548, or thymic inflammation, such as miR-125b and miR-146 [16]. Here, we showed that miR-29a/b1 seems also link to thymic inflammation. In mouse, if a decrease of miR-29a/b1 can optimized IFN-I signalization by modulating *Ifnar1* mRNA expression in TECs [5], it could also lead to *Ifn-β* mRNA expression in the context of EAMG, in addition to other pro-inflammatory cytokines such as *Il-6*, *Il-17a,* and *Il-21*. In the human thymus, a decreased expression of miR-29a-3p was observed in MG patients and also in TECs upon IFN-β treatment. Yet, it is not clear if the decreased expression of miR-29 subtypes in MG is either a consequence or a causative factor of the thymic inflammation. Anyhow, our results indicated that a reduction in miR-29 subtypes may contribute to the pathophysiological process involved in MG by favoring the emergence of pro-inflammatory Th17 cells.

## Data Availability

The datasets used and/or analyzed during the current study are available from the corresponding author.

## Abbreviations

AChR: Acetylcholine receptor
CFA: Complete Freund’s adjuvant
HET: Heterozygous
IFN: Interferon
miRNA: MicroRNA
MG: Myasthenia gravis
TEC: Thymic epithelial cell
WT: Wild-type
